# Structure of nucleosome-bound human PBAF complex

**DOI:** 10.1038/s41467-022-34859-5

**Published:** 2022-12-10

**Authors:** Li Wang, Jiali Yu, Zishuo Yu, Qianmin Wang, Wanjun Li, Yulei Ren, Zhenguo Chen, Shuang He, Yanhui Xu

**Affiliations:** 1 Fudan University Shanghai Cancer Center, Institutes of Biomedical Sciences, State Key Laboratory of Genetic Engineering and Shanghai Key Laboratory of Medical Epigenetics, Shanghai Medical College of Fudan University, Shanghai, 200032 China; 2 The International Co-laboratory of Medical Epigenetics and Metabolism, Ministry of Science and Technology, China, Department of Systems Biology for Medicine, School of Basic Medical Sciences, Shanghai Medical College of Fudan University, Shanghai, 200032 China; 3 Human Phenome Institute, Collaborative Innovation Center of Genetics and Development, School of Life Sciences, Fudan University, Shanghai, 200433 China; 4 The Fifth People’s Hospital of Shanghai, Shanghai Institute of Infectious Disease and Biosecurity, Shanghai Key Laboratory of Medical Epigenetics, and Institutes of Biomedical Sciences, Fudan University, Shanghai, 200032 China

**Keywords:** Chromatin remodelling, Cryoelectron microscopy

## Abstract

BAF and PBAF are mammalian SWI/SNF family chromatin remodeling complexes that possess multiple histone/DNA-binding subunits and create nucleosome-depleted/free regions for transcription activation. Despite previous structural studies and recent advance of SWI/SNF family complexes, it remains incompletely understood how PBAF-nucleosome complex is organized. Here we determined structure of 13-subunit human PBAF in complex with acetylated nucleosome in ADP-BeF_3_-bound state. Four PBAF-specific subunits work together with nine BAF/PBAF-shared subunits to generate PBAF-specific modular organization, distinct from that of BAF at various regions. PBAF-nucleosome structure reveals six histone-binding domains and four DNA-binding domains/modules, the majority of which directly bind histone/DNA. This multivalent nucleosome-binding pattern, not observed in previous studies, suggests that PBAF may integrate comprehensive chromatin information to target genomic loci for function. Our study reveals molecular organization of subunits and histone/DNA-binding domains/modules in PBAF-nucleosome complex and provides structural insights into PBAF-mediated nucleosome association complimentary to the recently reported PBAF-nucleosome structure.

## Abstract (web summary)

BAF and PBAF are SWI/SNF family chromatin remodeling complexes that create nucleosome-depleted regions for transcription activation. Here the authors report the structure of a 13-subunit human PBAF in complex with acetylated nucleosome in ADP-BeF_3_-bound state that provides structural insights into PBAF-mediated nucleosome association.

## Introduction

The adenosine triphosphate (ATP)-dependent chromatin remodeling complexes (also known as remodelers) regulate chromatin architecture by reorganizing nucleosome positioning and content^1,2^. SWI/SNF is the prototype chromatin remodeler possessing nucleosome sliding activity and unique histone ejection activity, and by which creates nucleosome-depleted or nucleosome-free regions (NDRs/NFRs) on gene promoters required for transcription initiation^3–5^. SWI/SNF and RSC complexes are two SWI/SNF family members in yeast and their counterparts in mammals are BRG1/BRM-associated factor (BAF) and polybromo-associated BAF (PBAF) complexes^6–8^, respectively. In line with their functional importance in gene regulation, BAF and PBAF are among the most frequently mutated complexes in cancer, as up to 20% of malignancies have alterations on coding genes of BAF/PBAF subunits^9–12^.

**Fig. 1:**
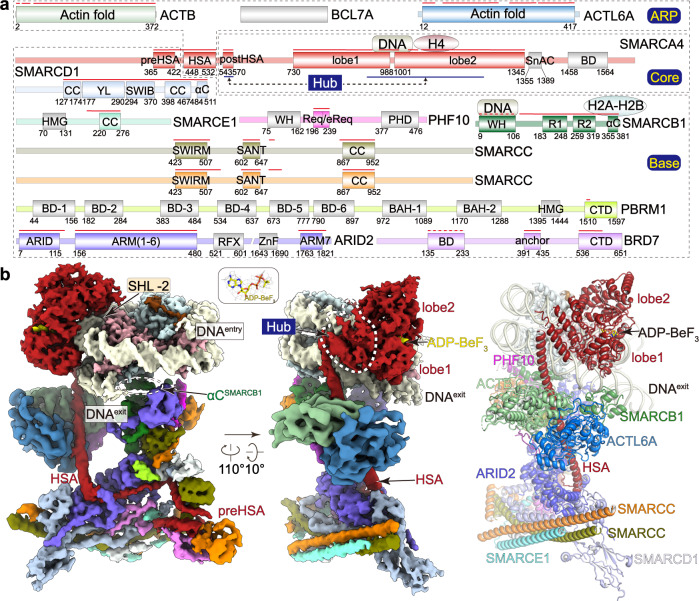
Cryo-EM structure of the nucleosome-bound PBAF. **a** Schematic diagram of the 13-subunit PBAF complex organized into the Base, ARP, and Core modules. Domains are shown with boundaries indicated with numbers. Histone/DNA-binding domains of indicated subunits are shown. Color scheme is used throughout figures if not elsewhere specified. The assigned regions are indicated with solid lines and the docked regions shown in dashed lines. **b** Composite cryo-EM map and structural model of PBAF-NCP in two different views. Close-up views of ADP-BeF_3_ is shown in cryo-EM map (mesh) and structural models (stick).

BAF and PBAF are megadalton multi-subunit complexes, which share a catalytic subunit SMARCA4 (also known as BRG1) and eight common auxiliary subunits, including ACTB, ACTL6A, BCL7A, SMARCB1 (BAF47), SMARCD1 (BAF60A), SMARCE1 (BAF57), and two SMARCC1/2 (BAF155 and BAF170, equivalent and termed SMARCC for simplicity) (Fig. 1a). The two complexes are distinguished by BAF-specific subunits ARID1A/B (BAF250A/B), DPF1/2/3 (BAF45B/D/C), and SS18 and PBAF-specific subunits ARID2 (BAF200), PHF10 (BAF45A), PBRM1 (BAF180), and BRD7. ARID1A/B and DPF1/2/3 in BAF are equivalent subunits of ARID2 and PHF10 in PBAF, respectively. PBRM1 and BRD7 exist exclusively in PBAF and have no equivalent subunits in BAF. Despite recent advances in structural studies of nucleosome-bound human BAF^13,14^, yeast RSC^15–17^, and yeast SWI/SNF^18^ complexes, structure of human PBAF remains unknown. Consistent with the difference in complex composition, BAF and PBAF exhibit distinct preference in genomic localization^19,20^ and functions^9–11,21^, suggesting a PBAF-nucleosome structure distinct from that of BAF-nucleosome. SWI/SNF family complexes consist of multiple (over 20 in PBAF) histone-binding and DNA-binding domains that are believed to facilitate genomic targeting and/or regulate remodeling activities of these complexes. However, it remains largely unknown how these histone/DNA-binding domains are organized within apo or nucleosome-bound SWI/SNF complexes. Here, we determined structure of nucleosome-bound PBAF showing multivalent nucleosome-binding pattern of human PBAF and provided structural basis for understanding the PBAF function in chromatin remodeling.

## Results

### Overall structure of the nucleosome-bound PBAF complex

We overexpressed the 13-subunit human PBAF complex in Expi293F suspension cells through co-transfection of plasmids containing full-length open reading frames (ORFs) of the catalytic subunit SMARCA4 and twelve auxiliary subunits (Fig. 1a, Supplementary Fig. 1a). The complex was purified to homogeneity for biochemical and structural analyses. In vitro chromatin remodeling assay showed that the purified PBAF converted a center-positioned nucleosome core particle (NCP) to three products, the end-positioned nucleosome, end-positioned H3-H4 tetrasome, and free DNA, indictive of its activity in sliding and ejection of nucleosome in a time-dependent manner (Supplementary Fig. 1b and c).

The chromatin substrates in cells of BAF/PBAF commonly contain acetylation at multiple sites of histone tails, which may regulate chromatin remodeling activity of SWI/SNF family complexes and facilitate their binding to nucleosome^21–23^. We reconstituted unmodified nucleosome and performed an in vitro acetylation reaction using a mixture of two predominate human histone acetyltransferases (HATs), p300 and SAGA acetyltransferase subcomplex (Supplementary Fig. 1d). The acetylation at a variety of histone lysine residues including H3K14 was detected by mass spectrometry (Supplementary Data 2), although the level of acetylation could not be accurately measured. Acetylation of histone H3 at residue K14 (H3K14), a representative histone acetylation, was validated by site-specific antibody. In vitro remodeling assay showed comparable activities of PBAF on un-acetylated and acetylated nucleosomes (Supplementary Fig. 1e).

The purified PBAF was incubated with the acetylated nucleosome (nucleosome or NCP hereafter) in 1:1 stoichiometry in the presence of ATP analog ADP-BeF_3_, followed by gradient fixation (Grafix) and cryo-EM sample preparation. Cryo-EM structure of PBAF-NCP was determined to overall resolution of approximately 4.4 Å (Supplementary Figs. 2, 3, Supplementary Table 1, Supplementary Movies 1, 2). The PBAF-NCP complex is organized into three modules including the Core, the actin-related proteins (ARP)^24^, the multi-subunit Base modules. Cryo-EM maps of the Base and Core modules were improved by focus refinement to near-atomic (3.4 Å to 4.1 Å) resolution and the ARP to 5.4 Å resolution. Structural models were built according to the cryo-EM maps with structure of BAF as reference 14 and aided by cross-linking mass spectrometry (XL-MS) analysis (Supplementary Fig. 4, Supplementary Data 1).

**Fig. 2:**
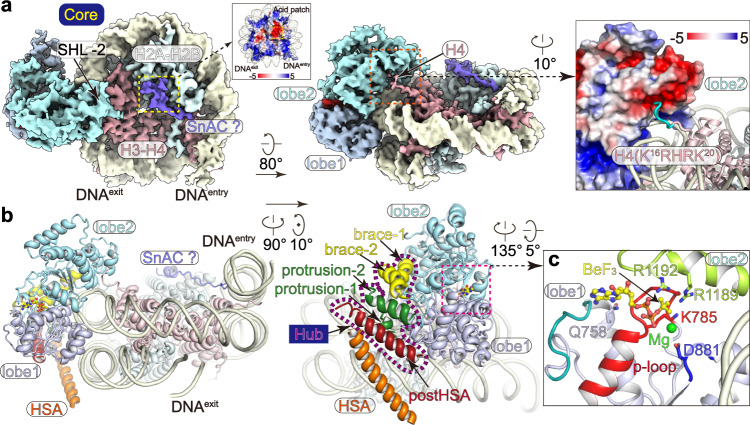
Structure of the nucleosome-bound ATPase. **a** Cryo-EM map of the Core module in two different views. Binding of an unassigned region (putative SnAC domain) to histone acidic surface is highlighted and binding of histone H4 tail to the acidic surface of ATPase lobe2 is shown in close-up view. **b** Structural model of the Core module with the Hub helices indicated. **c** Close-up view of the nucleotide-binding pocket with ADP-BeF_3_ and critical residues shown in sticks.

**Fig. 3:**
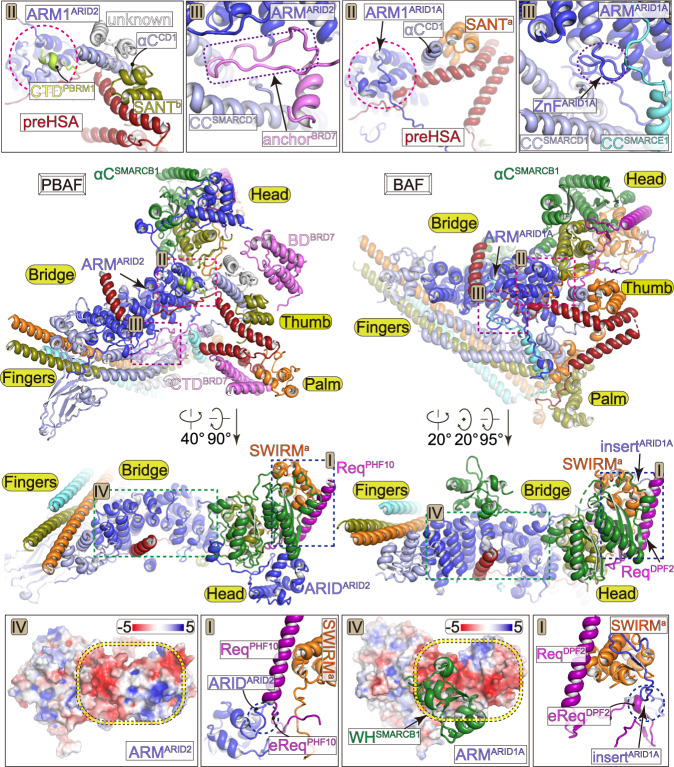
PBAF-specific organization of the Base module. Comparison of the Base modules of PBAF (left panels) and BAF (right panels) in two different views. Structural differences at region-I to region-IV are indicated on overall structure and shown in close-up views. The differences at equivalent positions are highlighted with dashed circles.

PBAF-NCP structure reveals a tripartite modular organization and is generally similar to the structures of nucleosome-bound human BAF^13,14^ and yeast SWI/SNF and RSC complexes^15–18^ (Fig. 1b, Supplementary Fig. 5). SMARCA4 serves as a central scaffold that involves formation of three modules. The Core module is formed by the nucleosome-bound SMARCA4 C-terminal portion, which consists of a regulatory Hub connecting helicase-SANT-associated (HSA) helix and ATPase, a nucleosome-bound ATPase motor (Figs. 1 and 2). An ATPase-associated density was observed on the surface of histone octamer (Supplementary Fig. 3). XL-MS analysis showed cross-links between K1365^SMARCA4^-K109^H2B^ and K1375^SMARCA4^-K96^H2A^, suggesting that this region might be derived from Snf2 ATP coupling (SnAC) domain (residues 1355–1389) of SMARCA4^25,26^. The ARP module is formed by ACTB-ACTL6A heterodimer and HSA helix of SMARCA4 that bridges the Core and Base. Cryo-EM map of ARP module reveals density of an unassigned region (Supplementary Fig. 3). The Base module accounts for the majority of molecular mass and is formed by the preHSA region of SMARCA4 and nine auxiliary subunits (Figs. 1 and 3).

**Fig. 4:**
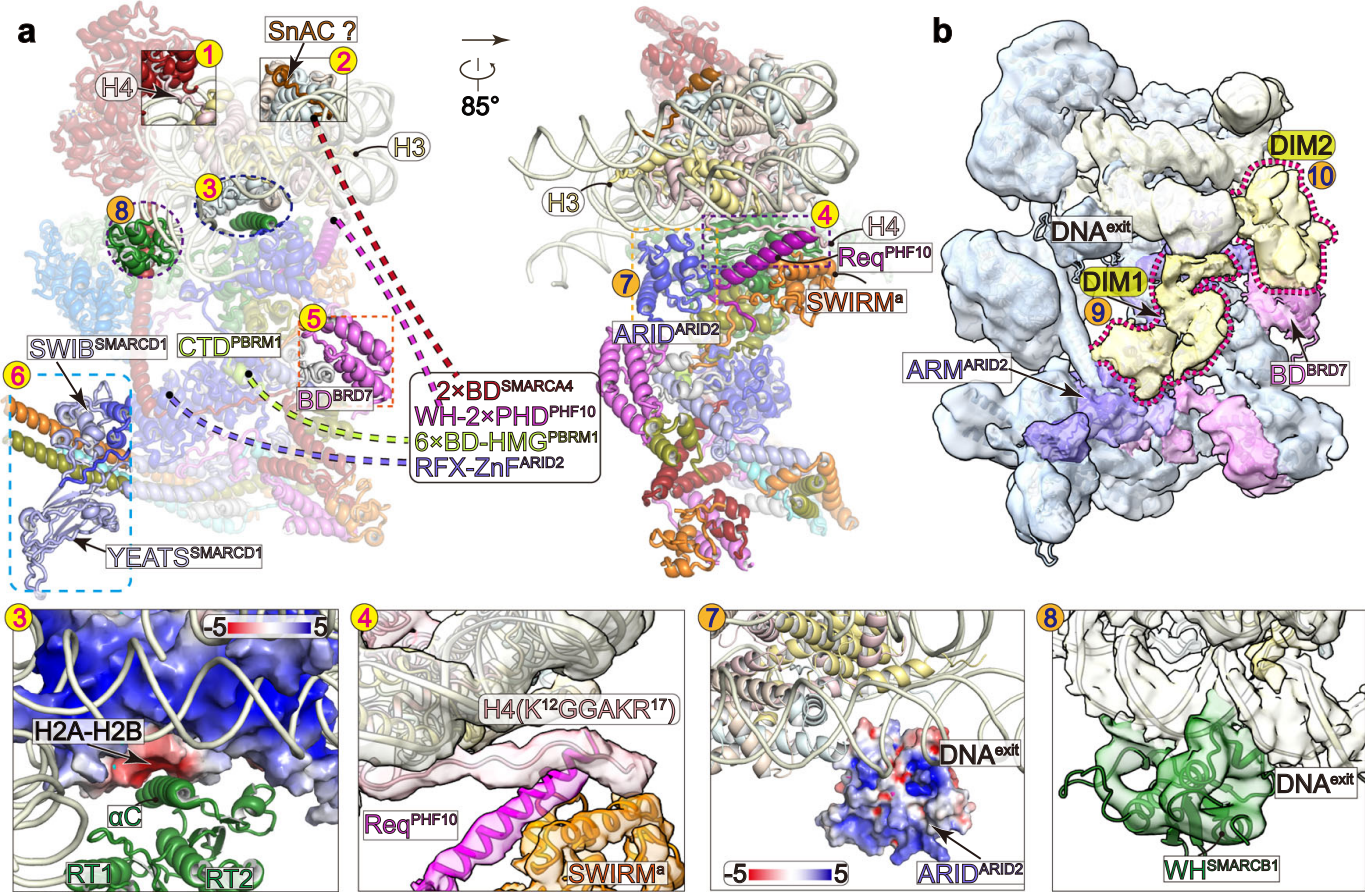
Histone/DNA-binding domains/modules. **a** Structural model and **b** cryo-EM map at low threshold of PBAF-NCP with the positions of histone/DNA-binding domains/modules indicated with numbers. Numbers 1 to 6 in yellow balls indicate the histone-binding domains and 7 to 10 in orange balls indicate the DNA-binding domains/modules. Invisible domains are connected with dashed lines to indicate their tethers with ordered regions. The bottom panels show close-up views of indicated contacts with involved regions shown in transparent cryo-EM map or electrostatic potential surface. Unassigned regions around exiting DNA, DIM1, and DIM2, are colored in yellowed and highlight with dashed lariats.

PBAF makes multiple contacts with nucleosome. Within the Core module, the ATPase stably grasps nucleosomal DNA at superhelical location (SHL) −2 and the interaction is buttressed by the tether between ATPase and histones (Figs. 1 and 2). Within the Base module, multiple DNA-binding and histone-binding domains/modules directly bind or are positioned near the nucleosome core particle (Figs. 1 and 4).

### The ATPase grasps nucleosomal DNA and the Hub couples the ATPase and Base

Structure of the ADP-BeF_3_-bound ATPase at near-atomic resolution shows network of interactions between the ATPase and ADP-BeF_3_, which results in a close conformation with the ATPase lobe1 and lobe2 grasping nucleosomal DNA (Fig. 2, Supplementary Fig. 6). As the mimetic *γ*-phosphate of ATP, BeF_3_ is stabilized by residues R1189 and R1192 of lobe2 and residue K785 within the P-loop of lobe1. The magnesium cation is coordinated by BeF_3_ and residue D881 of lobe1. Structural comparison of PBAF-NCP with nucleosome-bound ATPase of yeast Snf2 (PDB: 5Z3U)^27^ shows similar overall fold of the ATPase in ADP-BeF_3_-bound state, consistent with highly conserved catalytic mechanism. Compared to the isolated Snf2, the ATPase of PBAF slightly rotates and the Hub helices are displaced by up to 6 Å, likely resulted from the association of the Base through a regulatory Hub that connecting the HSA and ATPase.

The postHSA-containing Hub of SMARCA4 and HSA-containing ARP module are essential for the function of SWI/SNF complexes and cancer-associated gain-of-function mutations are enriched on the Hub^28–31^. However, structural organization of the Hub was not fully understood. The PBAF-NCP structure shows that the Hub is formed by five α helices derived from postHSA, protrusion1/2, and brace1/2 (Fig. 2b, Supplementary Fig. 6). Two protrusion helices associate with ATPase lobe1 on one side and postHSA helix on the other side. Two brace helices form a helix hairpin, which associates with the two ATPase lobes and packs against the protrusion helices. Such domain organization suggests that the Hub couples the motions of ATPase and HSA-associated ARP-Base and therefore regulates chromatin remodeling activity.

### PBAF-specific modular organization of the Base

The Base of PBAF consists of six BAF/PBAF-shared subunits including SMARCA4, SMARCB1, SMARCD1, SMARCE1, and two SMARCC, and four PBAF-specific subunits including PBRM1, BRD7, ARID2 (equivalent to BAF-specific ARID1A/B), and PHF10 (equivalent to BAF-specific DPF1/2/3) (Figs. 1, 3, Supplementary Fig. 7, Supplementary Movies 1 to 3). The Base module is divided into five submodules, including the Bridge that nucleates assembly of the Base, the Head that directly binds histone core particle, the Fingers formed by five-helix bundle and associated domains, the helical Thumb, and a split Palm. Consistent with the compositional similarity and differences, PBAF and BAF complexes share generally similar modular organization of the tripartite architecture. However, PBAF does exhibit PBAF-specific modular organization, distinct from that of BAF mainly at four regions, termed region-I to region-IV for simplicity.

### ARID2 serves as a scaffold for assembly of the Base

The central Bridge consists of a superhelical armadillo (ARM) repeat derived from the majority of ordered region in ARID2 (ARM^ARID2^), which covers residues 157–1817 and contains a structurally flexible insert (residues 480–1752) (Figs. 1, 3, Supplementary Fig. 7). Similar to that of BAF, the ARM^ARID2^ has seven ARM repeats (ARM1 to ARM7) arranged into a superhelical fold and serves as a rigid core to nucleate the Base formation through binding preHSA region of SMARCA4 and other Base subunits. ARM^ARID2^ and ARM^ARID1A^ exhibit compositional and conformational difference in ARM repeats and ARM-associated loops, generating distinct modular organization of PBAF and BAF at regions I, III, and IV (detailed below).

Within the Head, the Req helix of PHF10 (Req^PHF10^) packs against a groove of SWIRM^a^ (one of the two SWIRM domains of two SMARCC) and repeat domain 2 (RPT2) of SMARCB1, similar to the binding pattern of Req^DPF2^ in BAF-NCP structure^14^, consistent with highly conserved residues for the interaction (Fig. 3, Supplementary Figs. 8, 9). At the region-I, the N-terminal ARID domain of ARID2 (ARID^ARID2^) packs against the two RPT domains of SMARCB1 and an extension of Req domain (eReq) of PHF10. Specific incorporation of PHF10, instead of BAF-specific DPF1/2/3, in PBAF may collectively result from the presence of eReq^PHF10^-binding ARID^ARID2^ and the lack of an insert of ARID1A, which stabilizes DPF1/2/3-specific eReq in BAF (Supplementary Figs. 7, 8d, 9).

### PBAF-specific PBRM1 and BRD7

Around the region-II of the Thumb, the C-terminal α helix of SMARCD1 (αC^SMARCD1^) is surrounded by a helix of preHSA of SMARCA4, a SANT domain of one SMARCC (SANT^b^), and three short α helices (Fig. 3, Supplementary Fig. 7). One of the three helices binds the Bridge on the ARM1^ARID2^ whereas this contact would generate steric clash with an ARM1^ARID1A^-associated loop in BAF. By contrast, the lack of PBRM1 in BAF leads to direct binding of the Thumb and Bridge and a rotation of the Thumb to the Head compared to that in PBAF. Cryo-EM map at near-atomic resolution indicated that this α helix is derived from the C-terminal domain of PBAF-specific PBRM1 (CTD^PBRM1^, residues 1514–1524). The assignment is further supported by XL-MS analysis, showing cross-links between K1520 of PBRM1 and K622 of SMARCC SANT^b^ (Supplementary Figs. 3, 4).

PBRM1 exclusively exists in PBAF and has no equivalent subunit in BAF. Besides the CTD^PBRM1^, PBRM1 consists of six bromodomains (BD), a bromo-adjacent homology (BAH), and a high mobility group (HMG) domain that account for the majority of molecular mass but were not structurally observed in the cryo-EM map. A number of cancer-derived nonsense mutations resulted in truncations of PBRM1 that lack of CTD^PBRM1^ ^32^ (Supplementary Fig. 9b), consistent with the importance of CTD^PBRM1^ in assembly and function of PBAF complex.

Around the region-III, the Bridge and Fingers make direct contacts between ARM5-ARM7 and Fingers helix bundle (Fig. 3, Supplementary Figs. 7, 8). An anchor motif of BRD7 (anchor^BRD7^) inserts into the gap between the Bridge and Fingers and facilitates their interactions. By contrast, equivalent gap in BAF is occupied by two ARM^ARID1A^-associated loops coordinated by a zinc finger^14^, which are absent in ARID2. The C-terminal domain of BRD7 (CTD^BRD7^) binds two N-terminal helices of SMARCA4 and a SANT domain of the other SMARCC (SANT^a^), generating a split Palm submodule that associated with the Thumb. The lack of CTD^BRD7^ in BAF allows this submodule to form a full Palm around the Fingers end through binding SMARCC helices and Pillar helices derived from SMARCD1 and SMARCE1.

Unexpectedly, the winged helix domain of SMARCB1 (WH^SMARCB1^) associates with ARM5^ARID1A^ of the Bridge in BAF^14^ but was not observed in equivalent position in PBAF (Fig. 3, region-IV). By contrast, WH^SMARCB1^ in PBAF binds nucleosomal DNA at SHL + 6.5 (Fig. 4, described below). Distinct positioning of WH^SMARCB1^ may result from the difference in charge distribution of WH^SMARCB1^-binding surface of ARM^ARID1A^ and equivalent region in ARM^ARID2^, which result from their sequence difference.

### The placements of multiple nucleosome-binding domains/modules

PBAF consists of over 20 nucleosome-binding domains/modules (Fig. 1a) that are thought to be functionally important in PBAF-mediated chromatin remodeling^21^. However, only a few of these domains/modules were observed in previously reported structures of SWI/SNF complexes^13–18^. Cryo-EM structure of PBAF-NCP reveals the placements of six histone-binding domains/motifs (Figs. 1, 4, Supplementary Fig. 8), including the H2A-H2B heterodimer-bound αC^SMARCB1^ and a putative SnAC^SMARCA4^, the H4 tail-bound ATPase lobe2 and Req^PHF10^-SWIRM^SMARCC^ heterodimer, and histone-free BD^BRD7^ and YEATS-like domain of SMARCD1 (YEATS^SMARCD1^), and four DNA-binding domains/modules, including ARID^ARID2^ and WH^SMARCB1^, and two unassigned DNA-interaction modules (DIM1 and DIM2) around exiting DNA of the bound nucleosome.

### PBAF binds nucleosome on two H2A-H2B heterodimers and two H4 tails

Locally refined cryo-EM map of the Core shows that the catalytic subunit SMARCA4 stably engages with nucleosome (Figs. 2a, 4a). Apart from DNA-ATPase interaction, the acidic patch of ATPase lobe2 binds the N-terminal positively charged tail (K^16^RHRK^20^) of histone H4 (Fig. 4a, position-1) and unassigned region (putative SnAC domain) extends out of the ATPase domain and winds over the acidic patch of H2A-H2B heterodimer (Fig. 4a, position-2). This region extends to the nucleosomal DNA at SHL −6. The two nucleosome-SMARCA4 tethers may facilitate nucleosome-association of PBAF during DNA translocation, consistent with the known functions of K16/20 acetylation of histone H4^21^ in regulating activities of SWI/SNF complexes.

The positively charged helix αC^SMARCB1^ binds the acidic patch of the bottom H2A-H2B heterodimer and binding pattern is similar to that observed in BAF-NCP structure^14^ (Fig. 4, position-3). In PBAF and BAF complexes, the Core and Base are similarly connected by the H2A-H2B heterodimer-αC^SMARCB1^ and the Hub-HSA-ARP tethers (Supplementary Fig. 7). Some cancer-associated mutations are enriched on αC^SMARCB1^ (loss of function)^33^ and the Hub (gain of function)^30^, supporting their functional importance in both BAF and PBAF complexes.

Due to the differences in composition and arrangement of nucleosome-binding domains, PBAF and BAF exhibit distinct intermodular organization of the Core and Base (Fig. 4, Supplementary Fig. 7, Supplementary Movie 3). For example, compared to that of BAF, the Head of PBAF rotates toward nucleosomal DNA at +2, permitting the binding of the N-terminal tail of histone H4 to Req^PHF10^-SWIRM^SMARCC^ heterodimer (Fig. 4a, position-4, Supplementary Fig. 8b). The interaction is likely mediated by positively charged residues of H4 tail and a negatively charged patch of Req^PHF10^-SWIRM^SMARCC^ heterodimer. It remains further investigation whether this contact has any biological function.

### Positions of two putative histone-binding domains

We observed relatively weak cryo-EM map positioned near CTD^PBRM1^ within the Thumb (Fig. 4, position-5, Supplementary Fig. 2). XL-MS analysis showed cross-link between K119 of the bromodomain of BRD7 (BD^BRD7^) and K622 of SMARCC SANT^b^, suggesting that the density is derived from the BD^BRD7^ (Supplementary Fig. 4, Supplementary Data 1). Predicted structural model of BD^BRD7^ was placed into the map and the putative histone-binding site is about 70 and 50 Å away from the histone fold regions of H3 and H4, respectively (Supplementary Fig. 8). Thus, BD^BRD7^ is accessible to acetylated histone tails, ~30–40 residues in length, in line with its predicted function in binding of histone acetylation.

Within the Bridge, the SWIB domain of SMARCD1 is organized by four short helices and serves as a helical extension of the ARM repeat (Fig. 4, position-6, Supplementary Fig. 8a). The YEATS-like domain of SMARCD1 (YEATS^SMARCD1^) associates with SWIB^SMARCD1^ and the coiled-coil of SMARCD1 in the Fingers. YEATS^SMARCD1^ is organized into two parallel four-stranded β-sheets, as in other YEATS domains, but lacks a conserved acetyl-lysine binding pocket. As a peripheral domain, YEATS^SMARCD1^ is positioned by as far as 120 Å away from nucleosome core particle, suggesting its role independent of binding nucleosome. Nevertheless, the YEATS-like domain is strictly conserved among the snf12/Rsc6/SMARCD1 family proteins, which regulates expression of genes in stress response^34,35^.

### DNA-binding domains around the exiting DNA

Apart from the ATPase, additional DNA-binding domains were observed around the exiting DNA (Fig. 4, position-7). ARID domains of ARID1A/1B and ARID2 are predicted to bind DNA^36^ but not structurally observed in previous studies of SWI/SNF complexes. ARID^ARID2^ in the Head submodule has no direct contact with nucleosomal DNA but its positively charged surface faces toward DNA, leaving the possibility to make a direct DNA-binding if underwent conformational change.

Cryo-EM map of PBAF-NCP shows noticeable density associated with nucleosomal DNA at SHL + 6.5. Structural model of WH^SMARCB1^ fits this density well (Fig. 4, position-8, Supplementary Fig. 8c) and the placement was further supported by XL-MS (Supplementary Fig. 4). This modular organization suggests that the positively charged helix inserts into the major groove of DNA with a cluster of characteristic basic residues (R37/R40/K45/R46/R52/R53) positioned near phosphate groups for charge-charge interactions, exhibiting a DNA-binding pattern similar to previously proposed model^37^.

Cryo-EM map reveals an unassigned density of DIM1 connecting ARM helices of the Bridge and extranucleosomal DNA at 10-bp to 20-bp downstream of the exit site at SHL + 7, suggesting that DIM1 is possibly derived from the RFX-like DNA-binding domain (RFX) and/or zinc finger (ZnF) of ARID2 (Fig. 4b, position-9, Supplementary Fig. 8e). Another unassigned region (DIM2) associates with the BD^BRD7^ and makes contacts with extranucleosomal DNA (Fig. 4b, position-10, Supplementary Fig. 8e). This region is possibly derived from DNA-binding domains of nearby subunits, such as WH domain of PHF10, HMG domain of PBRM1, and C2H2 zinc finger of ARID2.

PBAF-DNA contacts are enriched around the exiting site of nucleosomal DNA, a characteristic feature distinct from that of BAF-NCP structure, in which neither equivalent DNA contact nor placement of DNA-binding domains was observed^14^. Cryo-EM map of nucleosome-bound RSC (yeast counterpart of PBAF) at low resolution showed that an unassigned DIM binds extranucleosomal DNA 20-bp to 40-bp downstream of SHL + 7 of nucleosome^15^ (Supplementary Fig. 8f). While DNA-binding pattern differs in PBAF and RSC, binding of exiting DNA may represent an evolutionarily conserved function in targeting PBAF/RSC to promoters^38–41^.

Apart from the observed histone-binding and DNA-binding domains/modules, PBAF also consists of domains/modules that are invisible in the cryo-EM map, including two bromodomains of SMARCA4, WH domain and two PHD domains of PHF10, and HMG and six bromodomains of PBRM1, which are tethered with the Core module, the Head and Thumb submodules, respectively, consistent with their predicted roles in binding DNA and modified histone tails and targeting chromatin for function (Fig. 4).

## Discussion

PBAF and BAF share identical subunits and equivalent complex-specific subunits, and therefore are commonly considered highly similar in structure and function. PBAF differs from BAF in its presence of multiple acetylation-binding bromodomains. However, our study unexpectedly shows marked structural difference in their modular organization of the Base and placement of DNA/histone-binding domains. PBAF-specific subunits, ARID2, PBRM1, PHF10, and BRD7 not only provide PBAF-specific nucleosome-binding domains but also alter the placements of some nucleosome-binding domains of the PBAF/BAF-shared subunits, such as WH^SMARCB1^ and SWIRM^SMARCC^. Consistent with their functional requirement, the majority of nucleosome-binding domains exist or associate with the Head and Thumb submodules, which are positioned near the nucleosome. Such multivalent nucleosome-binding pattern was not observed in previous studies^13–18^. The PBAF-NCP structure may provide a framework to further investigate whether, and if yes, how these nucleosome-binding domains work coordinately or redundantly in integrating chromatin marks for remodeling chromatin targets. It also remains further investigation whether BAF and PBAF function, such as ejection of nucleosome, in a similar manner or not, given the distinct composition and modular organization.

Cancer-derived mutations are also frequently observed in PBAF-specific subunits^32,42^ (Supplementary Fig. 9b). Missense mutations predominately occur on domains that are involved in complex assembly (binding of other subunits) or histone/DNA-binding domains, in line with the critical roles of these domains in PBAF function. Large number of nonsense mutations occur throughout PBRM1 and ARID2, consistent with the importance of CTD^PBRM1^ and ARM7^ARID2^ in organizing the Base module.

During preparation of this manuscript, a structure of human PBAF-NCP was published^43^. The complex was assembled with unmodified nucleosome and 12-subunit PBAF (lack of BCL7A). To improve complex behavior in structure determination, Yuan et al. removed the N-terminal region of SMARCA4 (residues 1–159), the first four bromodomains of PBRM1 (residues 1–630), and the internal region of ARID2 (residues 627–1591). Cryo-EM map at high resolution indeed favor the assignment of Base subunits, including CTD^PRMB1^ and BD^BRD7^. By contrast, we assembled the complex using acetylated nucleosome and human PBAF containing 13 full-length subunits. The generated cryo-EM map revealed YEATS^SMARCD1^ domain and additional PBAF-NCP contacts including DNA-WH^SMARCB1^, DNA-DIM1, DNA-DIM2, and histone H4 tail-Req^PHF10^-SWIRM^SMARCC^. Thus, our study provides complementary structural insights into multivalent interactions between PBAF and nucleosome and a framework for understanding PBAF functions in chromatin targeting and remodeling.

## Methods

### PBAF expression and purification

The thirteen full-length open reading frames (ORFs) of PBAF subunits were subcloned into modified pMLink vector containing no tag or the N-terminal Flag tag and 4 x Protein A tag followed by an HRV-3C cleavage site. All human PBAF subunits were co-transfected into suspension Expi293F cells using polyethylenimine (Polysciences). Cells were cultured for 72 h at 37 °C and harvested by centrifugation. For complex purification, all the steps were performed at 4 °C. Cells were disrupted in lysis buffer containing 50 mM HEPES pH 8.0, 300 mM NaCl, 5% (v/v) Glycerol, 0.2% (w/v) chaps, 2 mM MgCl_2_, 0.5 mM EDTA (Ethylenediaminetetraacetic Acid), 2 mM DTT (Dithiothreitol), 1 mM PMSF (Phenylmethylsulfonyl fluoride), 1 μg/mL Aprotinin, 1 μg/mL Pepstatin, 1 μg/mL Leupeptin for 30 min. Cell lysate was clarified by centrifugation at 38,000 × *g* for 30 min. The supernatant was incubated with IgG resin for 4 h and washed thoroughly with wash buffer containing 20 mM HEPES pH 8.0, 150 mM KCl, 5% (v/v) Glycerol, 0.1% chaps, 2 mM MgCl_2_, 2 mM DTT. After on-column digestion overnight, immobilized protein was eluted using wash buffer and further loaded onto an ion-exchange column (MonoQ 5/50 GL column, GE Healthcare) to achieve highly pure PBAF complex. The peak fractions corresponding to PBAF complex were collected and concentrated to ~3 mg/mL. The concentrated samples were used for subsequent biochemical and structural analyses.

### Preparation of nucleosomes

Canonical human histone H2A-H2B heterodimer and H3.1-H4 heterotetramer were separately co-expressed as soluble protein in Escherichia coli BL21 (DE3) cells as described previously^44^. In brief, cells were disrupted in lysis buffer containing 50 mM Tris-HCl (pH 8.0), 2 M NaCl, 5% (v/v) glycerol, 0.7 mM β-mercaptoethanol (β-ME), and then purified through ion-exchange chromatography. For histone octamer assembly, H2A-H2B heterodimer in 1.2-fold excess was mixed with H3.1-H4 heterotetramer and then incubated for 0.5 h at 4 °C, followed by a size exclusion chromatography (Superdex 200 10/300, GE Healthcare). Peak fractions were concentrated and used for nucleosome assembly.

DNA fragments for mononucleosome reconstitution were prepared by PCR amplification^45^. Three different mononucleosome DNA used in this study contained the Widom 601 positioning sequence^46^. Nucleosome 45N45 (N denotes nucleosome) consists of two flanking sequences of 45-bp in length. Nucleosome 0N90 consists of one flanking DNA with 90-bp in length. Nucleosome 15N51 consists of a 15-bp and a 51-bp flanking DNAs.

A center-positioned nucleosome 45N45 was assembled and used as a substrate in nucleosome remodeling activity. The DNA sequence of 45N45 is as below:

GCATCCCTTATGTGAGGTACCCTATACGCGGCCGCCCCGGATCCCCTGGAGAATCCCGGTGCCGAGGCCGCTCAATTGGTCGTAGACAGCTCTAGCACCGCTTAAACGCACGTACGCGCTGTCCCCCGCGTTTTAACCGCCAAGGGGATTACTCCCTAGTCTCCAGGCACGTGTCACATATATACATCCTGTTCCAGTGCCGGGCATGTATTGAACAGCGTTTAAACCGGTGCCAGT(the ‘601’ positioning sequence is underscored).

An end-positioned nucleosome 0N90 was assembled and used as the reference in nucleosome remodeling activity. The DNA sequence of 0N90 is as below:

CTGGAGAATCCCGGTGCCGAGGCCGCTCAATTGGTCGTAGACAGCTCTAGCACCGCTTAAACGCACGTACGCGCTGTCCCCCGCGTTTTAACCGCCAAGGGGATTACTCCCTAGTCTCCAGGCACGTGTCACATATATACATCCTGTTCCAGTGCCGGTGTCGCTTGGGTCCCGAGGTATTCAAGCTTATCGATACCTGCGACCACGAGGGGGGGTGCCGGGCATGTATTGAACAGC(the ‘601’ positioning sequence is underscored).

The nucleosome 15N51 was assembled and used for cryo-EM. The DNA sequence of 15N51 is as below:

ATCCTGGGGAATTCCCTGGAGAATCCCGGTGCCGAGGCCGCTCAATTGGTCGTAGACAGCTCTAGCACCGCTTAAACGCACGTACGCGCTGTCCCCCGCGTTTTAACCGCCAAGGGGATTACTCCCTAGTCTCCAGGCACGTGTCACATATATACATCCTGTTCCAGTGCCGGTGTCGCTTGGGTCCCGAGGATTACAAGCTTATCGATAGAT(the ‘601’ positioning sequence is underscored).

The 15N51 DNA fragment was inserted into the pUC57 vector, and the plasmid DNA was amplified in the *E. coli* strain DH5α. The 15N51 DNA fragment was excised from the plasmid DNA by EcoR V. The 45N45 and 0N90 DNA fragments were prepared using PCR amplification. All the nucleosomal DNA fragments were purified using ion-exchange chromatography (Source Q 5/5, GE Healthcare) and isopropanol precipitation. The purified DNA pellet was dissolved in 1 x TE buffer.

Nucleosome reconstitution was performed by mixing DNA with octamer at an equimolar ratio, with a linear salt gradient dialysis according to previously published research^47^. Finally, nucleosomes were dialyzed to 1 x HE buffer (10 mM HEPES pH 8.0, 0.1 mM EDTA). The nucleosomes can be immediately used for complex assembly and biochemical assay.

### In vitro chromatin remodeling assay

In vitro chromatin remodeling assay was performed using purified PBAF complex and nucleosome 45N45 as substrate. Nucleosome 45N45 (120 nM) was mixed with PBAF (80 nM) in buffer containing 20 mM Tris-HCl (pH 8.0), 60 mM KCl, 5 mM MgCl_2_, 0.1 mg/mL BSA, 5% (v/v) glycerol. The reactions were started with the addition of 1 mM ATP at 30 °C and stopped at different time points (0, 0.25, 0.5, 3, 5, 10, 30 min) by adding competitor plasmid (~1 μg) in excess, followed by further incubation for 30 min on ice. The reaction samples were analyzed by 5% native PAGE gel at 4 °C and run in 0.5 x Tris-Glycine buffer for 50 min at 180 V constant. The PAGE gels were stained with GelRed dye and visualized using the Tanon-2500 image system. Ejection of H2A-H2B heterodimer was validated by western blotting using antibody against histone H2B.

### Nucleosome acetylation and mass spectrometry validation

To generate acetylated nucleosome, we purified two histone acetyltransferases (HATs), p300 and SAGA acetyltransferase subcomplex containing KAT2A, STAF36, TADA2B, and TADA3L. The expression and purification procedures are essentially similar to that of PBAF. Nucleosome 15N51 was acetylated in reaction buffer containing 50 mM Tris-HCl (pH 8.0), 0.1 mM EDTA, 10% (v/v) glycerol, 1 mM DTT, 1 mM PMSF. Nucleosome (1.6 µM) was first incubated with increasing concentration of the mixture of HATs at 30 °C for 5 min, followed by the addition of 50 µM acetyl-CoA for another 30 min at 30 °C^22^. Western blotting was used to detect the acetylation efficiency of nucleosome. For large-scale preparation of the acetylated nucleosome, 15N51 nucleosome and HATs were incubated in 1:1 stoichiometry with the reaction performed as mentioned above. The two HATs were separated from the PBAF-NCP complex during Grafix.

Using mass spectrometry for analysis of the degree of nucleosome acetylation, we prepared 2 μg acetylated nucleosomes as described above. The samples were loaded onto the SDS PAGE gel (6%–16%) and then the protein slices were excised from the CCB (Coomassie brilliant blue)-stained gel. Excised slices were firstly destained twice with 200 μl of 50 mM NH_4_HCO_3_ and 50% acetonitrile and then dried twice with 200 μl of acetonitrile. The dried pieces of gels were incubated in ice-cold digestion solution (trypsin 12.5 ng/μl and 20 mM NH_4_HCO_3_) for 20 min and then transferred into a 37 °C incubator for digestion overnight. Finally, peptides in the supernatant were collected after extraction twice with 200 μl extract solution (5% formic acid in 50% acetonitrile). The peptide solution described above was dried under the protection of N_2_.

The tryptic peptides were dissolved in 0.1% formic acid (solvent A), directly loaded onto a reversed-phase analytical column (Acclaim PepMap C18, 75 μm × 25 cm). The gradient was comprised of an increase from 2 to 30% solvent B (0.1% formic acid in 98% acetonitrile) over 50 min, 30 to 50% in 5 min and climbing to 80% in 1 min then holding at 80% for the last 4 min, all at a constant flow rate of 300 nl/min on an EASY-nLC 1200 UPLC system. The peptides were subjected to NSI source followed by tandem mass spectrometry (MS/MS) in Orbitrap Exploris 480 MS coupled online to the UPLC. Spray voltage were set to 2.3 kV, funnel RF level at 50, and heated capillary temperature at 320 °C. For DDA experiments full MS resolutions were set to 60,000 at *m*/*z* 200 and full MS AGC target was 300% with an IT of 25 ms. Mass range was set to 350–1600. AGC target value for fragment spectra was set at 200% with a resolution of 15,000 and injection times of 50 ms and Top12. Intensity threshold was kept at 2E5. Isolation width was set at 1.6 m/z. Normalized collision energy was set at 30%. The tandem mass spectrometry analyses of the acetylated nucleosome samples were technically repeated by 3 times.

MS raw files were processed using PEAKS Online X (build 1.6.2022-01-07_161036, Bioinformatics Solutions Inc.). The data were searched against the swissprot human database consisting of reviewed canonical sequences (total entry 20332). Parent mass tolerance was set to 10ppm, with fragment mass tolerance of 0.02 Da. Specific tryptic cleavage was selected with allows for a maximum of 2 missed cleavages. For identification of all proteins, fixed modifications of carbamidomethylation (57.02 Da) on cysteine residues were specified. Variable modifications of oxidation (15.99 Da) on methionine and methylation on lysine were specified. The minimum peptide is 6 in length. The minimum number of unique peptides for protein identification is 2. Label-free quantification was also performed using the PEAKS Online X quantification module, allowing a mass tolerance of 20 ppm and quantifying all peptides that pass a 1% FDR threshold.

### Complex assembly and gradient fixation

The PBAF-NCP complex was cross-linked and purified using gradient fixation (Grafix)^48^. In brief, the purified PBAF and acetylated nucleosome 15N51 were mixed at a ratio of 1:1 for 30 min at 4 °C followed by the addition of 2 mM MgCl_2_, 0.5 mM ADP, 7 mM NaF, 1 mM BeSO_4_ and incubation by 15 min at 30 °C. The assembled sample was loaded onto a gradient generated from a glycerol light solution containing 15% (v/v) glycerol, 20 mM HEPES pH 7.0, 60 mM KCl, 2 mM MgCl_2_, 0.5 mM ADP, 7 mM NaF, 1 mM BeSO_4_, 2 mM DTT and a glycerol heavy solution containing 35% (v/v) glycerol, 20 mM HEPES pH 7.0, 60 mM KCl, 2 mM MgCl_2_, 0.5 mM ADP, 7 mM NaF, 1 mM BeSO_4_, 2 mM DTT and 0.01% (v/v) glutaraldehyde. The sample was subjected to ultracentrifugation at 247,600 × *g* for 15 h in an SW41Ti swinging-bucket rotor (Beckman) at 4 °C. The peak fractions of the cross-linked PBAF-NCP complex were concentrated and dialyzed overnight against a buffer containing 20 mM HEPES pH 7.0, 60 mM KCl, 2 mM MgCl_2_, 0.5 mM ADP, 7 mM NaF, 1 mM BeSO_4_, 2 mM DTT, followed by cryo-EM grids preparation.

### Cryo-EM sample preparation

For negative staining EM grid preparation, samples (5 µL at a concentration of ~0.06 mg/mL) were applied onto glow-discharged copper grids supported by a continuous thin layer of carbon film for 60 s before negative staining by 2% (w/v) Uranyl Acetate solution at room temperature. The grids were prepared in the Ar/O_2_ mixture for 15 s using a Gatan 950 Solarus plasma cleaning system with a power of 35 W. The negatively stained grids were loaded onto a Thermo Fisher Scientific Talos L120C microscope equipped with a Ceta CCD camera and operated at 120 kV at a nominal magnification of ×92,000, corresponding to a pixel size of 1.58 Å on the specimen.

For cryo-EM grid preparation, samples (4 μL at a concentration of ~0.6 mg/mL) were applied to freshly glow-discharged Quantifoil R1.2/1.3 holey carbon grids. After incubation for 5 s at 6 °C and 100% humidity, the grids were blotted for 1 s with blot force 2 in a Thermo Fisher Scientific Vitrobot Mark IV and plunge-frozen in liquid ethane at liquid nitrogen temperature. The grids were prepared in the H_2_/O_2_ mixture for 60 s using a Gatan 950 Solarus plasma cleaning system with a power of 5 W. The ø 55/20 mm blotting paper is made by TED PELLA used for plunge freezing.

### Cryo-EM data collection

The cryo-EM grids were loaded onto a Thermo Fisher Scientific Titan Krios transmission electron microscope operated at 300 kV for data collection. Cryo-EM images were automatically recorded by a post-GIF Gatan K3 Summit direct electron detector in the super-resolution counting mode using Serial-EM with a nominal magnification of ×64,000 in the EFTEM mode, which yielded a super-resolution pixel size of 0.667 Å on the image plane, and with defocus values ranging from −1.0 to −2.5 μm. Each micrograph stack was dose-fractionated to 40 frames with a total electron dose of ~50 e^−^/Å^2^ and a total exposure time of 3.6 s. 9156 micrographs of PBAF-NCP were collected for further processing.

### Image processing

Movie stacks were corrected for drift and beam-induced motion correction by MotionCor2^49^ with binned 2-fold to a calibrated pixel size of 1.334 Å/pixel, which generated drift-corrected summed micrographs with and without electron-dose weighting. The defocus values were estimated by Gctf^50^ from non-dose-weighted summed images. Other procedures of cryo-EM data processing were performed within RELION v3.0^51,52^ and cryoSPARC v3^53^ using the dose-weighted micrographs.

Particles were automatically picked and subjected to reference-free 2D classification, yielding a total of 2,071,055 particles. The particles were further subjected to the 3D classifications. The classes with good quality consisted of 254,834 particles and were subsequently subjected to the 3D classification with Base module mask. And 92,526 particles were selected from good 3D classes, which were used for 3D classification in cryoSPARC v3. A final set of 37,528 homogeneous PBAF-NCP complex particles were selected to perform a final 3D reconstruction in cryoSPARC, yielding a reconstruction of PBAF-NCP complex at 4.4 Å resolution. Local refinement focused on the Core module with mask could reconstitute the Core module at 3.4 Å resolution. To improve the map quality of the Base module and the ARP module, the signal of Core module was subtracted from two classes of 3D classification containing 81,287 particles. The subtracted particles were further subjected to 2D classification, yielding 76,554 particles after clearance. A further 3D classification by applying mask for the ARP module and the Base module resulted in a clean dataset containing 31,995 particles. The resulting particles were refinement in cryoSPARC, yielding a reconstruction of ARP and Base module at 4.2 Å resolution. Local refinement focused on the ARP module and the Base module with mask generated reconstructions of the ARP module and the Base module at 5.4 Å and 4.1 Å, respectively.

All reported resolutions are based on the gold-standard Fourier shell correlation (FSC) = 0.143 criterion. The GSFSC curves were corrected for the effects of a soft mask with high-resolution noise substitution. All cryo-EM maps were sharpened by applying a negative B-factor estimation in cryoSPARC Sharpening Tools. All the visualization and evaluation of the 3D volume map were performed within UCSF Chimera or UCSF ChimeraX^54^, and the local resolution variations were calculated using cryoSPARC.

### Model building and structure refinement

The structures of RSC complex (PDB: 6TDA)^15,16,55^, (PDB: 6KW4)^16^, and structure of BAF complex (PDB: 6LTJ)^14^ were used as initial structural templates, which were fitted into the cryo-EM maps by rigid-body fitting using UCSF Chimera followed by iterative rounds of manual adjustment and rebuilding in COOT^56^. The model was finalized by rebuilding in ISOLDE^57^ followed by refinement in Phenix^58^ with secondary structure and geometry restraints using the cryo-EM maps. Overfitting of the model was monitored by refining the model in one of the two half maps from the gold-standard refinement approach and testing the refined model against the other map. Statistics of the map reconstruction and model refinement can be found in Supplementary Table 1. The final structural model was validated using Phenix. Map and model representations in the figures and movies were prepared by PyMOL (https://pymol.org/), UCSF Chimera or UCSF ChimeraX.

### Cross-linking and mass spectrometry analysis

The Cross-linking Mass Spectrometry (XL-MS) analysis was performed as previously described^59^. The purified PBAF complex (0.45 μM) was incubated with nucleosome at a ratio of 1:1 in the presence of ADP-BeF_3_ followed by cross-linking MS analyses. The PBAF-NCP complex was incubated with DSS (250 μM) at 25 °C with shaking at 500 rpm (ThermoMixer) for 1 h. Reaction was terminated by adding 20 mM ammonium bicarbonate (Sigma). The cross-linked sample was precipitated with cooled acetone and dried in a speed vac. The pellet was dissolved in 8 M Urea, 100 mM Tris-HCl pH 8.5, followed by TCEP reduction, iodoacetamide (Sigma) alkylation, and trypsin (Promega) digestion overnight at 37 °C using a protein/enzyme ratio of 50:1 (w/w). Tryptic peptides were desalted with Pierce C18 spin column (GL Sciences) and separated in a proxeon EASY-nLC liquid chromatography system by applying a step-wise gradient of 0–85% acetonitrile (ACN) in 0.1% foricacid. Peptides eluted from the liquid chromatography were directly electrosprayed into the mass spectrometer with a distal 2 kV spray voltage. Data-dependent tandem mass spectrometry (MS/MS) analyses were performed on Thermo Q-Exactive instrument in a 60-min gradient. The acquired raw data files were processed with pLink2 software^60^ and the results were visualized using the xiNET online server^61^.

### Reporting summary

Further information on research design is available in the Nature Portfolio Reporting Summary linked to this article.

## Supplementary Information

Supplementary Information

Description of additional Supplementary File

Supplementary Movie 1

Supplementary Movie 2

Supplementary Movie 3

Supplementary Data 1

Supplementary Data 2

Reporting Summary

## Data Availability

The data that support this paper are available from the corresponding authors upon request. The Cryo-EM map and atomic coordinate have been deposited in the EMDB with accession code of EMD-33684 and PDB with accession code of 7Y8R. Mass spectrometry results of acetylated nucleosome and XL-MS have been deposited to PRoteomics IDEntifications (PRIDE) Archive database with the accession number of PXD036708. Source data are provided with this paper.

## Source data

Source Data
