# Supplementary material for: Structure of nucleosome-bound human PBAF complex
Source: Nat Commun. 2022 Dec 10;13:7644. doi: 10.1038/s41467-022-34859-5 (PMC9741621; doi:10.1038/s41467-022-34859-5)
Supplement: Supplementary file 1 — Supplementary Information [file 41467_2022_34859_MOESM1_ESM.pdf]

Supplementary Information For

Structure of nucleosome-bound human PBAF complex

Li Wang[1,2,3]*, Jiali Yu[1]*, Zishuo Yu[1]*, Qianmin Wang[1]*, Wanjun Li[1], Yulei Ren[1], Zhenguo Chen[1,4]†, Shuang He[1]†, and Yanhui Xu[1,2,3]†

[1]Fudan University Shanghai Cancer Center, Institutes of Biomedical Sciences, State Key Laboratory of Genetic Engineering and Shanghai Key Laboratory of Medical Epigenetics, Shanghai Medical College of Fudan University, Shanghai 200032, China.

[2]The International Co-laboratory of Medical Epigenetics and Metabolism, Ministry of Science and Technology, China, Department of Systems Biology for Medicine, School of Basic Medical Sciences, Shanghai Medical College of Fudan University, Shanghai 200032, China.

[3]Human Phenome Institute, Collaborative Innovation Center of Genetics and Development, School of Life Sciences, Fudan University, Shanghai 200433, China

[4]The Fifth People's Hospital of Shanghai, Shanghai Institute of Infectious Disease and Biosecurity, Shanghai Key Laboratory of Medical Epigenetics, and Institutes of Biomedical Sciences, Fudan University, Shanghai 200032, China

* These authors contributed equally to this work.

† To whom correspondence should be addressed. E-mail: xuyh@fudan.edu.cn, hes@fudan.edu.cn, and zhenguochen@fudan.edu.cn

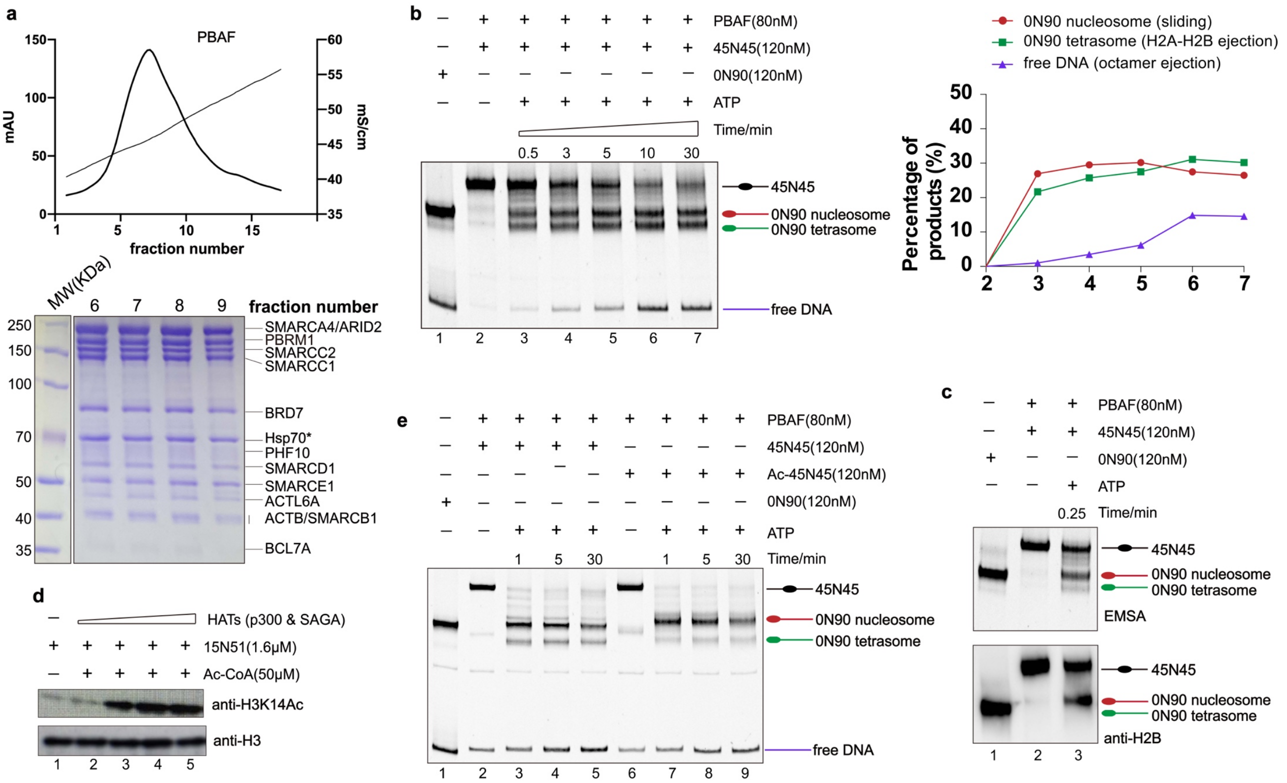

Supplementary Figure 1. Protein purification and remodeling activities of PBAF complex.

(a) Purification of PBAF complex. Profile of ion-exchange purification of the 13-subunit PBAF complex. Peak fractions were subjected to SDS-PAGE followed by Coomassie blue staining. (**b**) In vitro chromatin remodeling assay shows nucleosome sliding and ejection activities of PBAF. Reconstituted nucleosome 0N90 serves as a reference for an end-positioned nucleosome, the product of chromatin sliding reaction. (**c**) The generated 0N90 H3-H4 tetrasome represents the product of H2A-H2B ejection with the ejection of H2A-H2B dimer confirmed using antibody against histone H2B. Free DNA represents the product of histone octamer ejection. (**d**) In vitro acetylation of nucleosome by increasing concentration of a mixture of two acetyltransferases, p300 and SAGA acetyltransferase subcomplex. The level of acetylation was detected using antibody against acetylated histone H3K14. (**e**) Chromatin remodeling assay of nucleosomes with and without acetylation. All the experiments above were repeated at least three times.

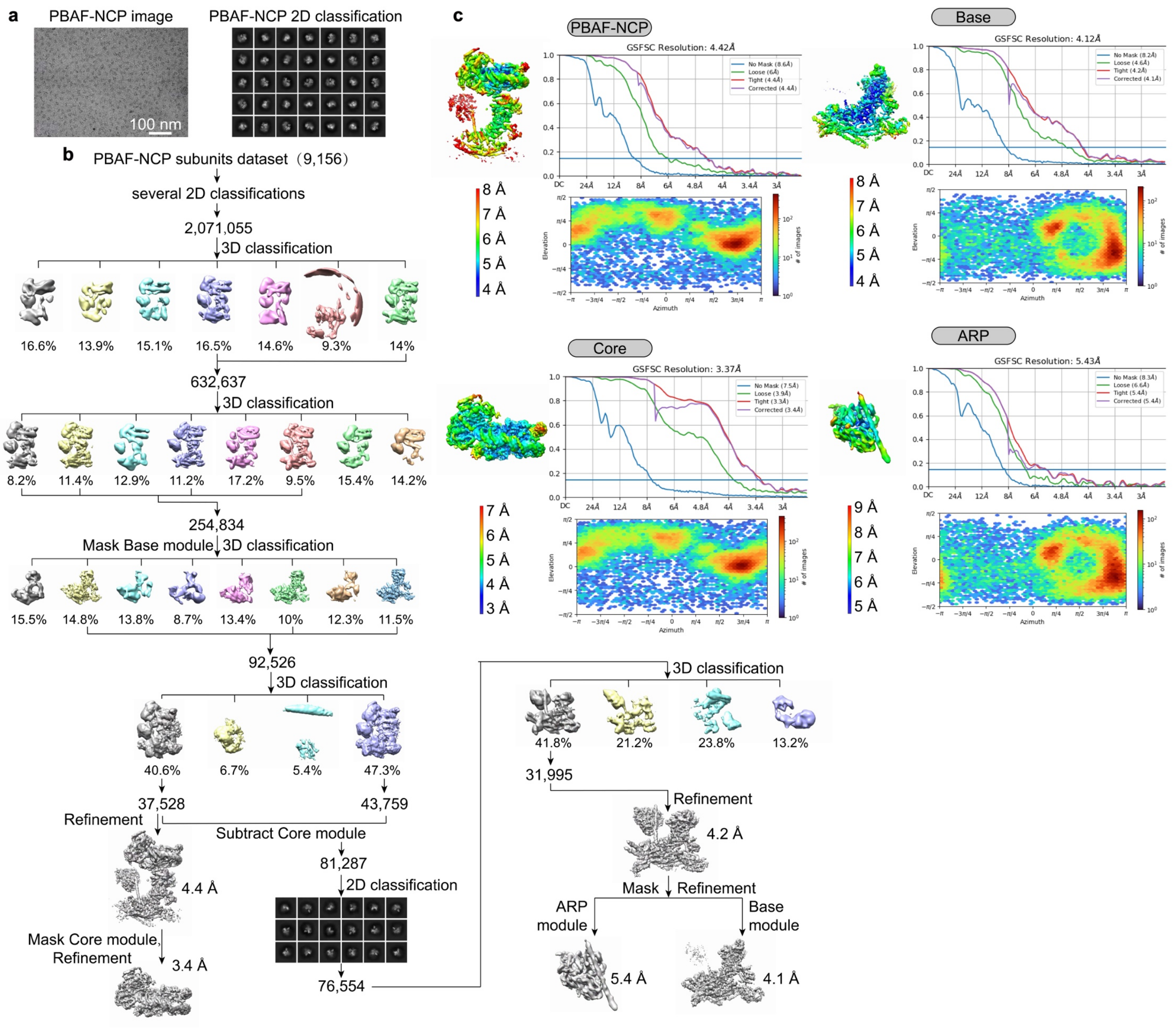

Supplementary Figure 2. Data collection and image processing of PBAF-NCP complexes.

(**a-b**) Representative cryo-EM images, 2D classification from at least three times repeatedly PBAF-NCP complex. (b) and flow-charts of the cryo-EM image processing (b) of PBAF-NCP sample. (**c**) Local resolution estimation, GSFSC curves and direction distribution of the cryo-EM reconstructions of whole complex and the Core, Base and ARP modules.

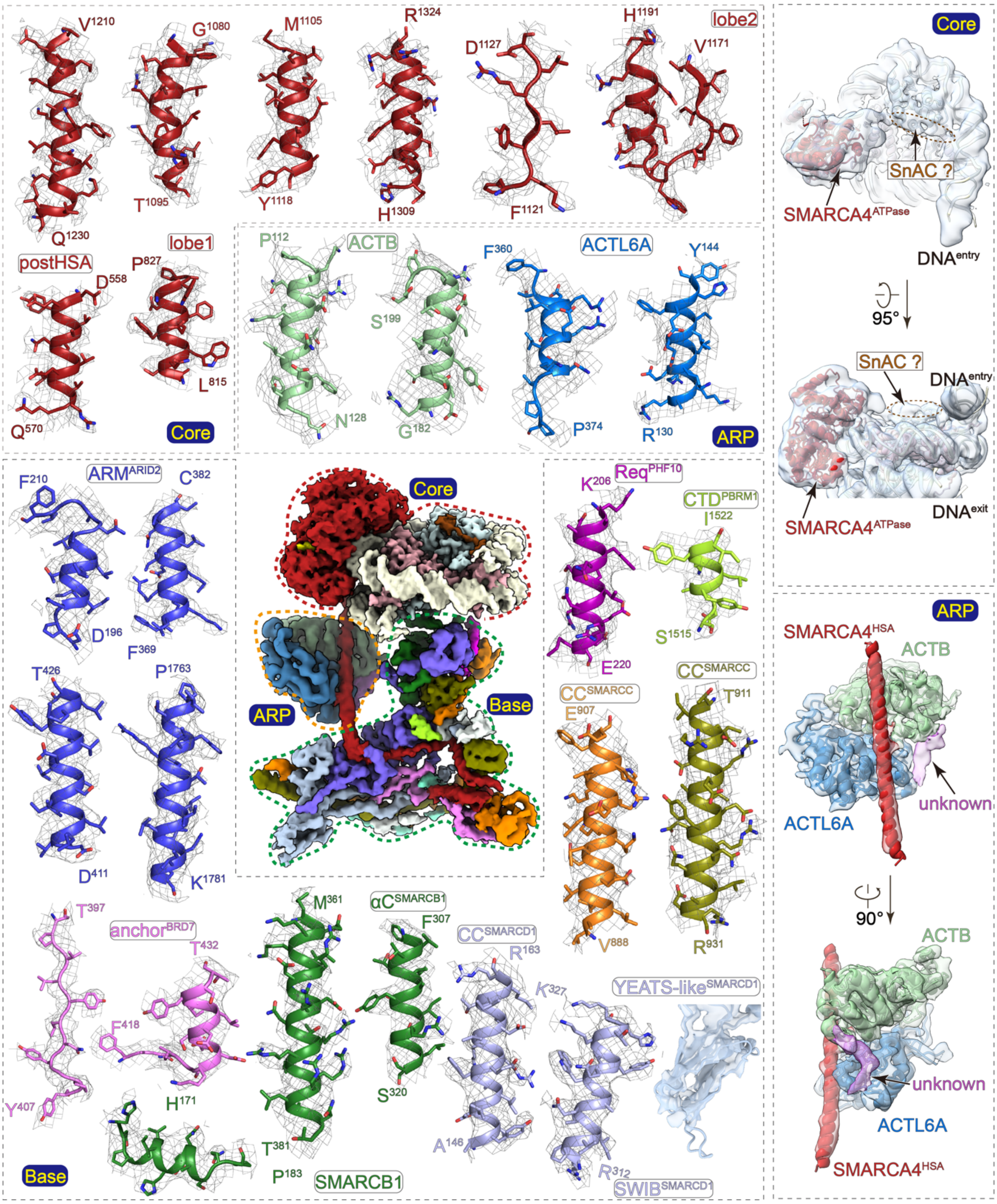

Supplementary Figure 3. Cryo-EM map and structural model.

Composite cryo-EM map of the Core module (3.4 Å), Base module (4.1 Å), and ARP module (5.4 Å). Indicated regions of PBAF subunits are shown in close-up views. Structural models shown in sticks representation are well covered by cryo-EM maps in meshes, supporting that the model was built correctly. Note that PBRM1 isoform 7 was used in our study. Right panel shows unassigned cryo-EM density above histone octamer and that within the ARP module.

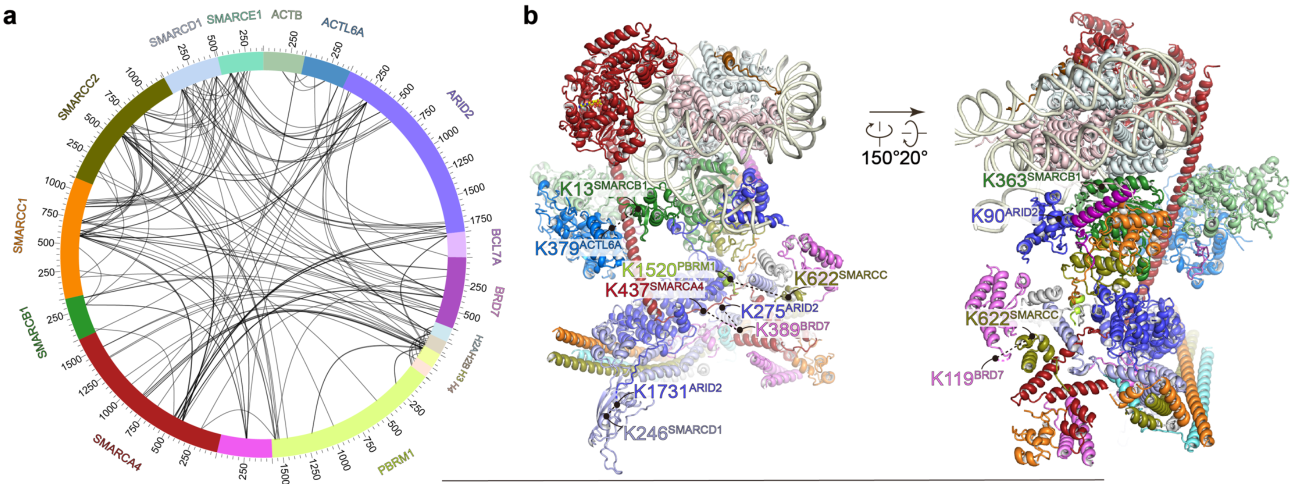

Linked site	Total spec	Distance	Reference Distance
K437^{SMARCA4}-K389^{BRD7}	9	–	K437^{SMARCA4}-D391^{BRD7}(21 Å)
K496^{SMARCA4}-K15^{BCL7A}	4	–	K496^{SMARCA4}-K30^{BCL7A}(30 Å)
K1365^{SMARCA4}-K109^{H2B}	2	9 Å	–
K1375^{SMARCA4}-K96^{H2A}	3	17 Å	–
K119^{BRD7}-K622^{SMARCC}	1	–	E130^{BRD7}-K622^{SMARCC}(15 Å)
K389^{BRD7}-K275^{ARID2}	1	–	D391^{BRD7}-K275^{ARID2}(21 Å)
K13^{SMARCB1}-K379^{ACTL6A}	1	26 Å	–
K363^{SMARCB1}-K90^{ARID2}	1	23 Å	–
K1520^{PBRM1}-K622^{SMARCC}	1	33 Å	–
K246^{SMARCD1}- K1731^{ARID2}	6	–	K246^{SMARCD1}- G1747^{ARID2}(15 Å)

Supplementary Figure 4. Cross-linking mass spectrometry.

(**a**) Schematic representation of inter-subunit cross-links within the PBAF-NCP complex in the presence of ADP-BeF_3. Intramolecular cross-links were omitted for simplicity. (**b**) crosslinks are listed in the table and the crosslinked residues are shown on the structural model. In the table, linked site shows crosslinks, distance measures the distance between Cα atoms of crosslinked residues, and reference distance measures the distance between structurally modeled residues (if the crosslinked residues are not modeled in our structure). Confident distance should be less than 30 Å according to the linker length.

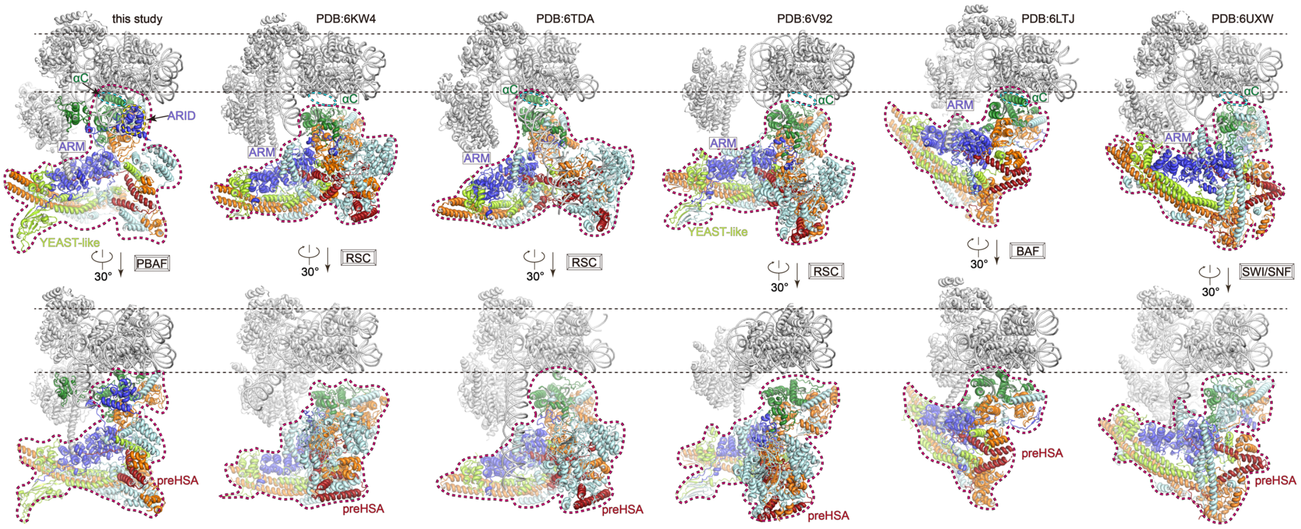

Supplementary Figure 5. Structural comparison of nucleosome-bound human PBAF and other SWI/SNF family complexes.

Two different views of the structures of nucleosome-bound human PBAF (this study), yeast RSC (PDB ID: 6KW4) [1], yeast RSC (PDB ID: 6TDA) [2], yeast RSC (PDB ID: 6V92) [3], human BAF (PDB ID: 6LTJ) [4], and yeast SWI/SNF (PDB ID: 6UXW) [5]. The structures are shown with nucleosome in a similar orientation for comparison. Equivalent subunits are colored in the same color scheme.

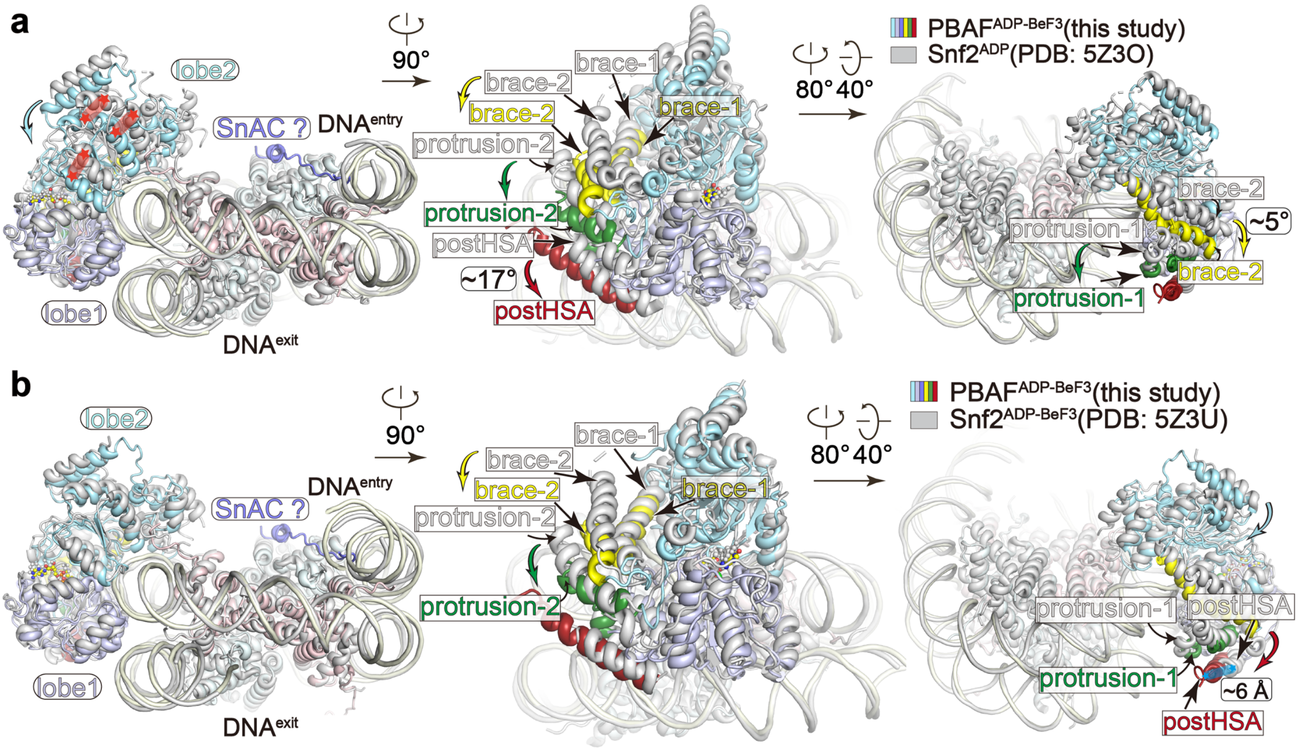

Supplementary Figure 6. Nucleosome-bound ATPase in PBAF complex and isolated Snf2.

Structural comparison of the nucleosome-bound ATPase in PBAF complex (ADP-BeF_3-bound) and isolated Snf2 ATPase [6] in the ADP-bound (**a**) and ADP-BeF_3-bound (**b**) states, respectively. The structures are shown with nucleosome superimposed with structural differences indicated with arrows. Structure of Snf2-NCP is colored in grey and that of PBAF-NCP is colored as indicated.

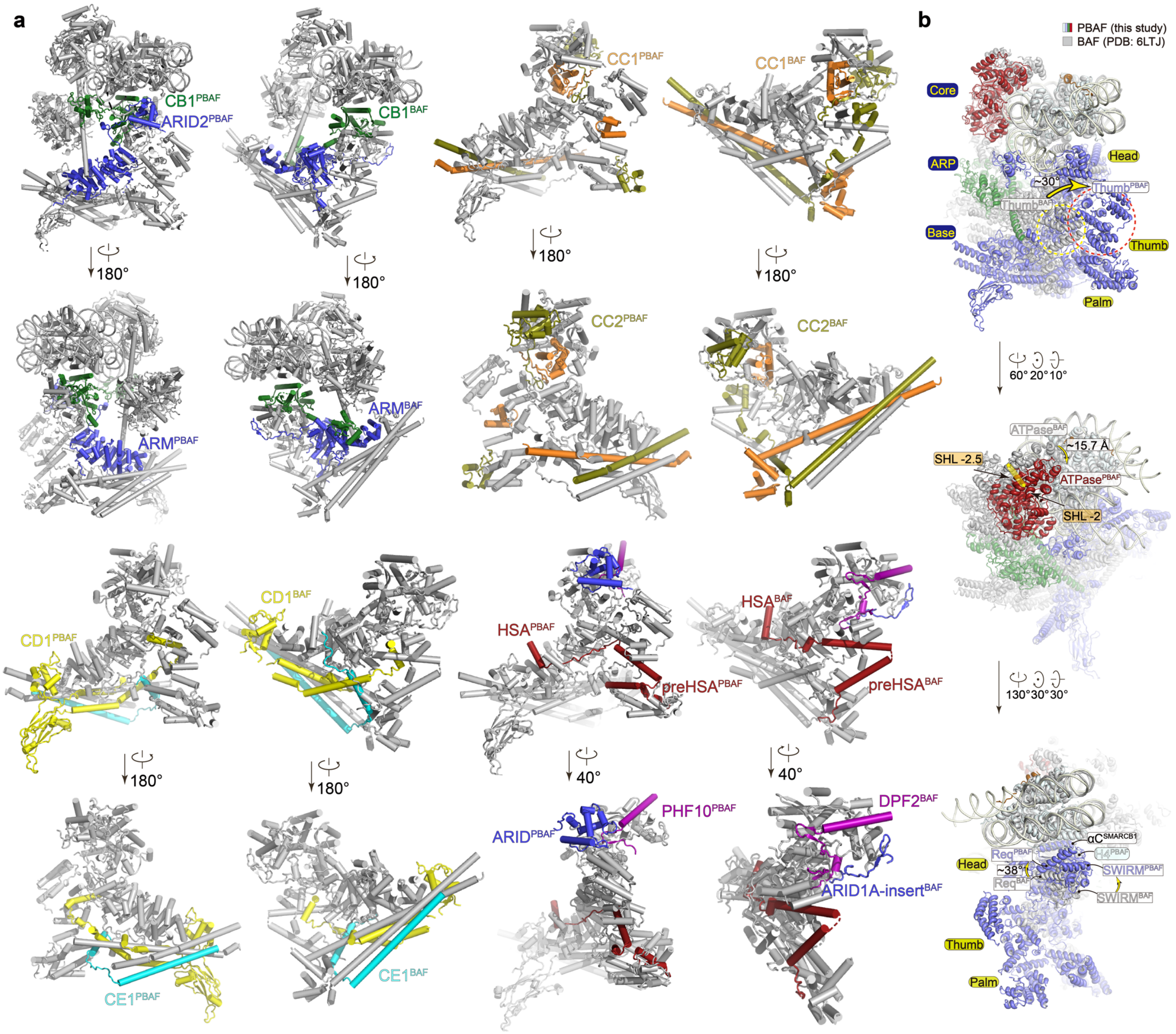

Supplementary Figure 7. Structural differences in PBAF-NCP and BAF-NCP.

(**a**) Structural comparison of PBAF-NCP (left panels) and BAF-NCP (right panels) structures with each subunit highlighted for comparison. (**b**) Superimposition of the two structures in three different views. Structural differences are indicated with arrows.

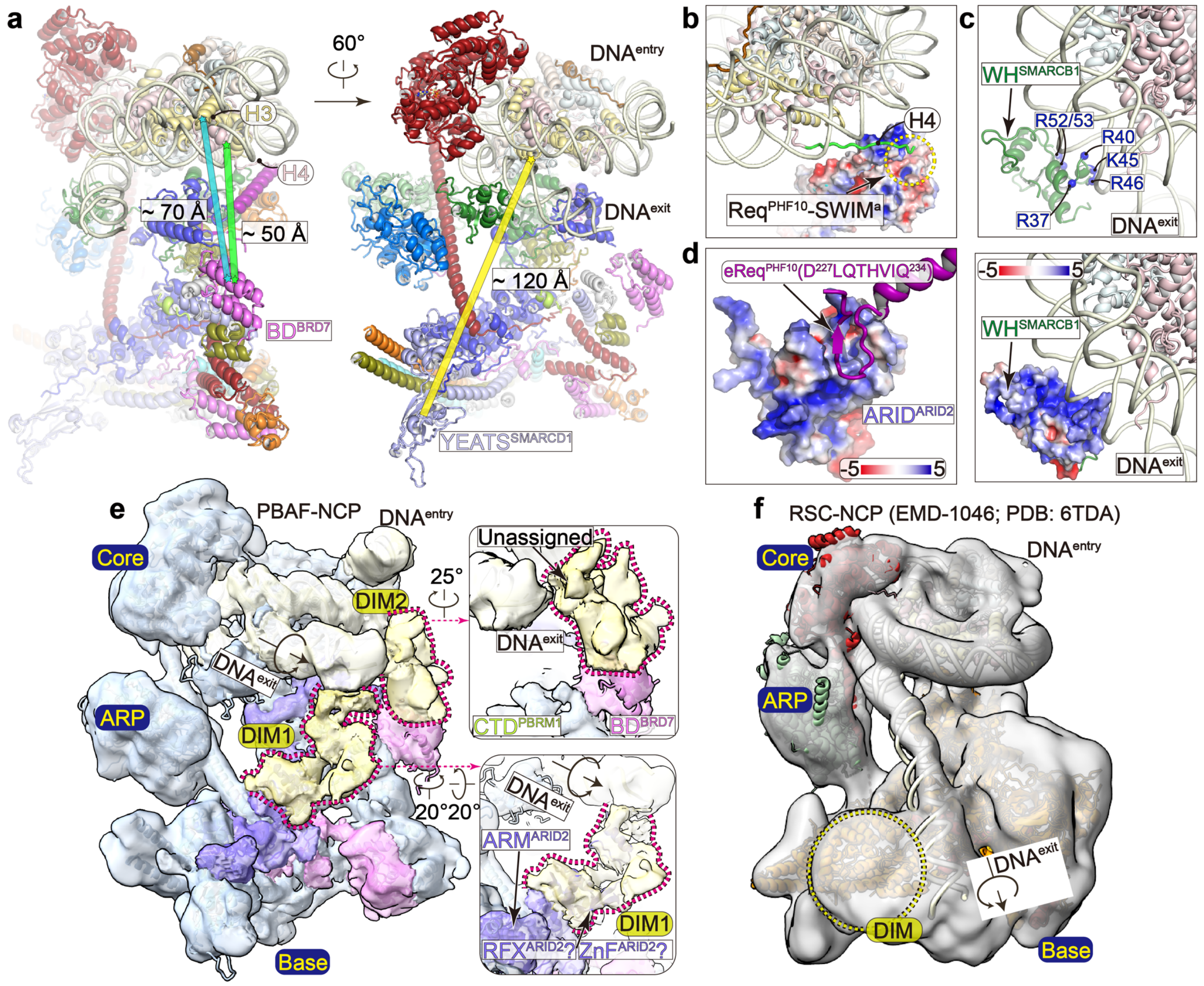

Supplementary Figure 8. Positions of histone/DNA-binding domains in PBAF-NCP.

(**a**) Overall structural model of PBAF-NCP in two different views. Distances between the bromodomain of BRD7 and histone fold domains of the nearest histone H3 and H4 are indicated in the left panel. Distance between YEATS-like domain of SMARCD1 and histone octamer is indicated in the right panel. (**b**) Close-up view of the contacts between histone H4 tail (shown in cartoon) and Req^{PHF10}-$SWIRM^{SMARCC}$ dimer. The acidic surface of Req^{PHF10}-$SWIRM^{SMARCC}$ is shown in electrostatic potential surface. (**c**) Close-up view of the interaction between $WH^{SMARCB1}$ and nucleosomal DNA. Positively charged residues of $WH^{SMARCB1}$ are indicated with blue balls in the upper panel. Positively charged surface of $WH^{SMARCB1}$ is indicated with electrostatic potential surface in the lower panel. (**d**) Interaction between $ARID^{ARID2}$ and PHF10. (**e**) Cryo-EM map of PBAF-NCP at low threshold shows unassigned regions of DIM1 and DIM2 that bind extranucleosomal DNA. Predicted structural model of RFX domain of ARID2 could be placed in the density of DIM1. (**f**) Cryo-EM map of RSC-NCP shows interaction between an unassigned DIM region and extranucleosomal DNA.

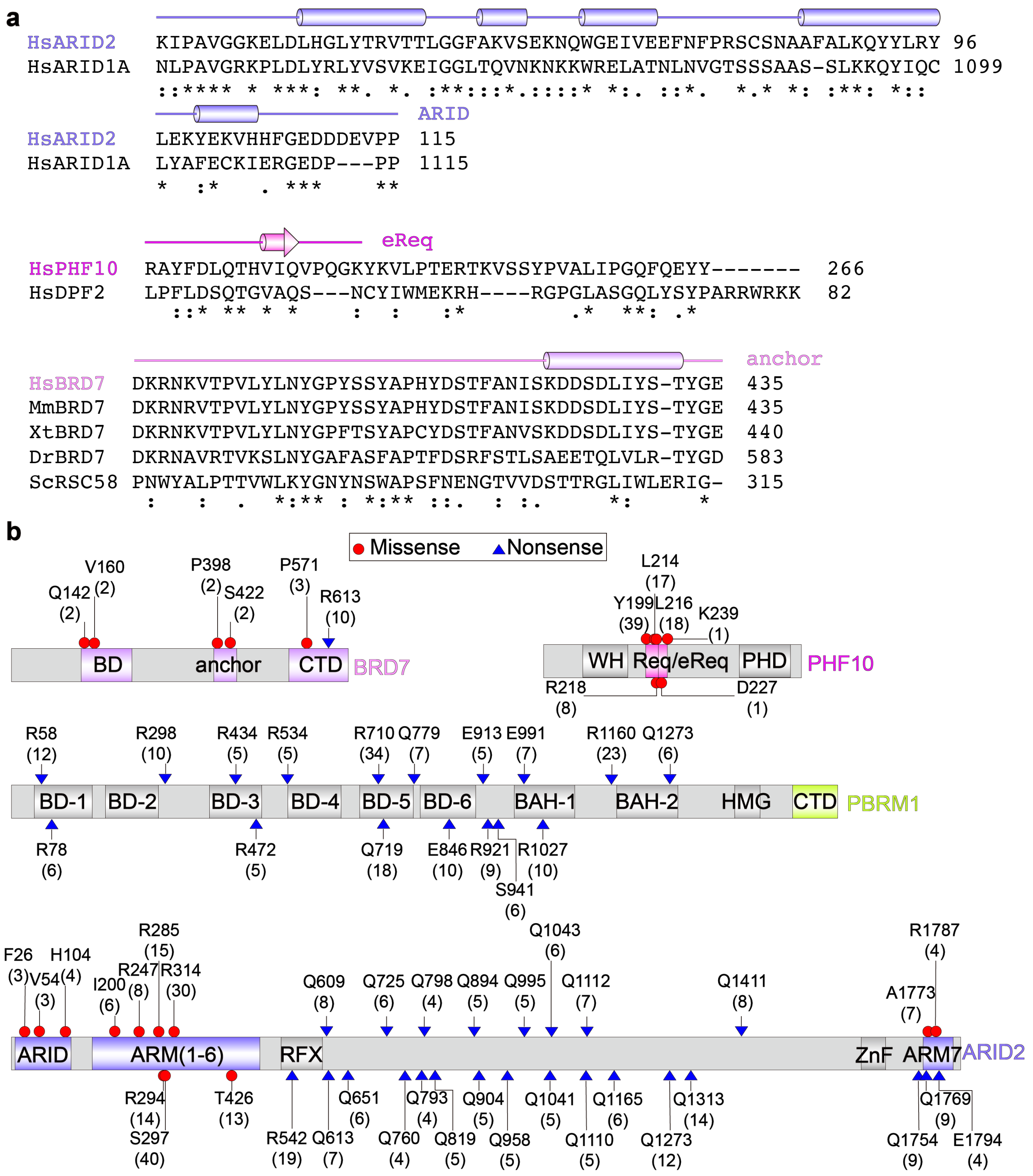

Supplementary Figure 9. Sequence analysis of PBAF-specific organization and cancer-derived mutations.

(**a**) Sequence analyses of PBAF-specific subunits with critical regions shown for comparison. (**b**) Cancer-derived mutations of PBAF-specific subunits.

Supplementary Table 1. Statistics of cryo-EM data collection and refinement.

	**PBAF-NCP** (EMDB-33684) (PDB-7Y8R)	**Base Module**	**ARP Module**	**Core Module**
**Data collection and processing**				
Magnification	64,000 x	64,000 x	64,000 x	64,000 x
Voltage (kV)	300	300	300	300
Electron exposure ($e^{-}/Å^2$)	50	50	50	50
Defocus range (μm)	-1.0 ~ -2.5	-1.0 ~ -2.5	-1.0 ~ -2.5	-1.0 ~ -2.5
Pixel size (Å)	1.334	1.334	1.334	1.334
Symmetry imposed	C1	C1	C1	C1
Initial particle images (no.)	2,071,055	2,071,055	2,071,055	2,071,055
Final particle images (no.)	43,759	31,995	31,995	43,759
Map resolution	4.4	4.1	5.4	3.5
FSC threshold	0.143	0.143	0.143	0.143
Map resolution range (Å)	4.0-8.0	4.0-8.0	5.0-9.0	3.0-7.0
**Refinement**				
Model resolution (Å)	4.43			
FSC threshold	0.143			
Model composition				
Non-hydrogen atoms	43,226			
Protein residues	4,581			
Nucleotide residues	330			
Ligands	1× ADP, 1× Mg, 1× BeF_3			
B factors ($Å^2$)				
Protein	104.10			
Nucleotide	90.25			
Ligand	20.04			
R.m.s deviations				
Bond lengths (Å)	0.004			
Bond angles (°)	0.813			
Validation				
MolProbity score	1.57			
Clash score	4.44			
Poor rotamers (%)	0.05			
Ramachandran plot				
Favored (%)	94.97			
Allowed (%)	5.00			
Disallowed (%)	0.02			

Supplementary References:

1 Ye, Y. *et al.* Structure of the RSC complex bound to the nucleosome. *Science* **366**, 838-843, doi:10.1126/science.aay0033 (2019).

2 Wagner, F. R. *et al.* Structure of SWI/SNF chromatin remodeller RSC bound to a nucleosome. *Nature* **579**, 448-451, doi:10.1038/s41586-020-2088-0 (2020).

3 Patel, A. B. *et al.* Architecture of the chromatin remodeler RSC and insights into its nucleosome engagement. *eLife* **8**, doi:10.7554/eLife.54449 (2019).

4 He, S. *et al.* Structure of nucleosome-bound human BAF complex. *Science* **367**, 875-881, doi:10.1126/science.aaz9761 (2020).

5 Han, Y., Reyes, A. A., Malik, S. & He, Y. Cryo-EM structure of SWI/SNF complex bound to a nucleosome. *Nature* **579**, 452-455, doi:10.1038/s41586-020-2087-1 (2020).

6 Li, M. *et al.* Mechanism of DNA translocation underlying chromatin remodelling by Snf2. *Nature* **567**, 409-413, doi:10.1038/s41586-019-1029-2 (2019).
