# Supplementary material for: Structure of nucleosome-bound human PBAF complex
Source: Nat Commun. 2022 Dec 10;13:7644. doi: 10.1038/s41467-022-34859-5 (PMC9741621; doi:10.1038/s41467-022-34859-5)
Supplement: Supplementary file 2 — Description of additional Supplementary File [file 41467_2022_34859_MOESM2_ESM.pdf]

Descriptions of Additional Supplementary Files

File name: Supplementary Movie 1

Description: Composite cryo-EM map and structural model of PBAF-NCP.

File Name: Supplementary Movie 2

Description: Cryo-EM map of PBAF-NCP at low threshold showing unassigned regions of DIM1 and DIM2.

File name: Supplementary Movie 3

Description: Structural comparison of PBAF-NCP (colored) and BAF-NCP (grey) with nucleosome superimposed.

File name: Supplementary Data 1

Description: Cross-linking mass spectrometry dataset of PBAF-NCP

File name: Supplementary Data 2

Description: Histone lysine acetylated residues detected by mass spectrometry
